# Nosologomania: DSM & Karl Jaspers' Critique of Kraepelin

**DOI:** 10.1186/1747-5341-4-10

**Published:** 2009-07-23

**Authors:** S Nassir Ghaemi

**Affiliations:** 1 Mood Disorders Program, Mood Disorders Clinic, Department of Psychiatry, Tufts Medical Center, 800 Washington Street, Box 1007, Boston, MA, 02111, USA

## Abstract

Emil Kraepelin's nosology has been reinvented, for better or worse. In the United States, the rise of the neo-Kraepelinian nosology of DSM-III resuscitated Kraepelin's work but also differed from many of his ideas, especially his overtly biological ontology. This neo-Kraepelinian system has led to concerns regarding overdiagnosis of psychiatric syndromes ("nosologomania") and perhaps scientifically ill-founded psychopharmacological treatment for presumed neo-Kraepelinian syndromes. In the early 20^th ^century, Karl Jaspers provided unique insights into Kraepelin's work, and Jaspers even proposed an alternate nosology which, though influenced by Kraepelin, also introduced the concept of ideal types. Jaspers' critique of Kraepelin may help us reformulate our current neo-Kraepelinian nosology for the better.

## Review

### Introduction

When Emil Kraepelin laid out his vision of psychiatric nosology at the turn of the twentieth century, he was met with plaudits but also opposition. The voices of opposition would gain ground after Kraepelin's death until the late twentieth century, when his views were reborn in the American neo-Kraepelinian school of psychiatry, influential in the revolutionary revision of the American nosological system, DSM-III, in 1980. Another contemporary critique of Kraepelin's nosology, by Karl Jaspers, has been underappreciated, however. In this paper, I review the evolution of Kraepelin's nosology in the United States, and examine Jaspers' critique of Kraepelin's work, especially the ideal type method, so as to improve our current neo-Kraepelinian nosology.

### The psychopharmacology revolution

After Kraepelin's initial popularity, his system was largely eclipsed, especially in the US, by the rise of the psychoanalytic movement [1], where (in the 1940s–1970s) there was a reversion to the nineteenth century insanity model, relabeled the neurosis-psychosis continuum [2]: everyone, patients or not, sick or healthy, fell on that continuum somewhere; the disease tradition in medicine was not considered central to psychiatry [1].

A turning point that led to the rebirth of Kraepelin's nosology began in the 1950s, when lithium was introduced for mania, chlorpromazine for schizophrenia, and imipramine for depression [3]. These medications were increasingly used and validated in clinical trials, and by the 1970s, a practical problem led to reassessment of the Freudian consensus on the neurosis-psychosis model [4]. If lithium worked specifically for mania, tricyclic antidepressants for depression, and phenothiazines for schizophrenia, then it would seem that Kraepelin's nosology was no longer therapeutically irrelevant. Despite later experience that these agents are relatively nonspecific [3], their apparent specificity in the 1950s–1970s led to enthusiasm for linking them to Kraepelin's nosology. The birth of psychopharmacology suggested that, at least for pharmacological treatment as well as for regulatory approval and drug-marketing [2], and surely also for service-reimbursement purposes, Kraepelin's schema seemed quite serviceable [4].

The "neo-Kraepelinians," many of whom were American or European researchers impressed by this process, began to test Kraepelin's nosology using new psychometric methods with demonstrable reliability and utility [1]. (In the US, headquartered at the Washington University in St. Louis, this group was famously led by Eli Robins and Samuel Guze). In the US-UK study of 1970 [5], researchers showed that American psychiatrists diagnosed many conditions they saw in New York as schizophrenia, based on loose definitions of the neurosis-psychosis continuum, while British psychiatrists in London diagnosed few conditions as schizophrenia (using Kraepelinian criteria), instead identifying most other conditions as mood or anxiety disorders. Specifically, comparing New York to London, schizophrenia was diagnosed in 65% vs 34%, and "depressive psychoses" in 7.2% vs. 32.8%, respectively. These were the largest differences; UK psychiatrists also diagnosed personality disorders more, though still infrequently, than New York psychiatrists (8.4% vs. 0.8%). When rediagnosed using standardized diagnostic criteria (the Present State Examination), only 32% of the American sample met schizophrenia definitions, as did 26% of the London sample; affective illness was more common (36.4% in New York, 47.2% in London) and personality disorders uncommon (2.4% in New York vs. 4.4% in London). Of those diagnosed with schizophrenia in New York, 53% were misdiagnosed, of which 71.3% had mood disorders. In London, schizophrenia was less frequently misdiagnosed (36.5%), but again mood disorders were the usual missed diagnoses (73.2%). In my reanalysis of those data using confidence intervals and relative risks, schizophrenia was misdiagnosed 46% more frequently in New York than London, mostly missing mood disorders (RR = 1.46, 95% confidence intervals 1.07, 2.01). The American approach seemed extremely broad, and the new medications suggested that making finer distinctions was practically important.

Though some have suggested that the rise of the neo-Kraepelinian DSM-III occurred independently of the psychopharmacology revolution [1], contemporaries have observed that the two seemed to play off each other at the time (Ross J. Baldessarini MD, Frederick K. Goodwin MD, personal communications, March 2006). Indeed, I suggest that the psychopharmacology revolution laid the groundwork for the neo-Kraepelinian restoration.

### The rise of DSM-III

Gerald Klerman, who coined the term 'neo-Kraepelinian' [6], outlined the benefits of DSM-III as follows:

First, it embodies the concept of multiple disorders, reaffirming psychiatry's acceptance of the modern medical model of disease.

Second, and for the first time, an official nomenclature has incorporated operational criteria with exclusion and inclusion criteria...based on manifest descriptive psychopathology rather than on presumed etiology – psychodynamic, social, or biological. This reliance on descriptive rather than etiological criteria does not represent an abandonment of the ideal of modern scientific medicine that classification and diagnosis should be by causation. Rather, it represents a strategic mode of dealing with the frustrating reality that, for most of the disorders we currently treat, there is only limited evidence for their etiologies....

Third, DSM-III underwent field testing for reliability. Never before have practitioners of a medical specialty participated in a test of the reliability of their nomenclature....

Fourth, a multiaxial system was introduced to accommodate the diverse aspects of our patients' existence.

Fifth, there is implicit in the creation of DSM-III...the necessity for change – the push for DSM-IV is already apparent. The changes that appear in DSM-IV should be determined by the state of evidence rather than the assertions of competing ideological camps [7].

Klerman addresses the issue of the uniqueness of individual patients well:

Most American psychiatrists, trained in the Meyerian tradition, have been reluctant to rely on diagnostic distinctions because of the acknowledged diversity of human attributes. Furthermore, in making clinical decisions about patients, diagnosis – reliable or not, valid or not – is only one criterion in decision-making....A diagnosis alone is not sufficient to account for all decision making; other attributes of the individual are crucial in the clinical context. No diagnostic system can encompass all these multiple attributes. The development of the multiaxial system represents a novel attempt to acknowledge this clinical experience....For scientific investigation in medicine and psychiatry, the unit of investigation is the disorder, while for clinical practice the unit is the individual [7].

One continues to hear this criticism today. Klerman's comments make it clear that the developers of DSM-III were not unaware of this potential problem. Klerman makes the distinction between diagnosis and clinical decision-making. A diagnosis is necessary, but not sufficient, for decision-making, he says. But practice today often consists of listing symptoms, making a diagnosis, and then prescribing a usually pharmacological treatment, without any further considerations. Perhaps with managed care insurance is relevant, when time limits (imposed by others or by oneself for financial gain) lead to less emphasis on individual differences among persons.

In a debate with Klerman, the psychoanalyst Robert Michels made a famous critique that centered on the lack of attention to subjective psychological states in the neo-Kraepelinian approach:

I hope that whatever happens in this debate, we do not lose all that is of value in American psychiatry that does not fit in 300 pages of operational criteria....In constructing a nosology, we must first decide whether we want to define the domain of the discussion as that which we can describe precisely or that which we believe to be relevant. DSM-III opts for the former. If this criterion for DSM-III had been employed in 1850, we would not have developed the concept of dementia praecox or schizophrenia by 1950; the initial formulation was too unreliable. The heterogeneity of our field is a potent argument for including, not excluding, a variety of concepts and ideas when developing nosology [7].

One definition of scientific method in nosology is the use of empirically testable hypotheses [8]. However, some hypotheses are certainly very difficult, if not impossible, to test empirically. Those related to subjective states, as in psychiatry, are especially difficult to test objectively. What should we do with these subjective states, and the hypotheses which involve them? Should we simply exclude them from the field of psychiatry, or perhaps include them but deny them any scientific validity (instead asserting that they are value judgments, as Kendler does) [8]? If we exclude them, we are in danger of creating what Michels calls 'mindless psychiatry.' If we say nothing or little about subjective states, psychiatry as a field would seem to lose a great deal of relevance to what patients experience and for which they want assistance [9].

Michels also coined the term 'invisible college' to describe the neo-Kraepelinian school in St. Louis that was heavily involved in preparing DSM-III [7]. The phrase has a slightly sinister flair to it, suggesting something of a conspiracy, though if there was a conspiracy, it was likely of events rather than of persons (though the protagonists in this drama, such as Robins, personalized this conflict with their opponents; R. Baldessarini MD, personal communication, March 2006). There is no evidence of an explicit conspiracy to start using drugs instead of psychoanalysis; to the contrary, Spitzer sought to mollify the interests of psychoanalysis, as he had to do to get the revision through the APA assembly [10].

### Karl Jaspers' earlier critique of Kraepelin

Karl Jaspers, in his 1913 magnum opus, *General Psychopathology *[11], provided an earlier critique of Kraepelin's work which was perhaps less appreciated by international psychiatry (and certainly practically unknown in the United States).

While critical, Jaspers was also appreciative of Kraepelin. In the Appendix (written in the 1940s) to *General Psychopathology*, he specifically also links Kraepelin's work to the incipient rise of psychopharmacology:

Kraepelin was responsible for one of the most fruitful lines of research, the investigation of the whole life-history of the patient. He...laid the foundations for psycho-pharmacology....But Kraepelin's basic conceptual world remained a somatic one which in the company of the majority of doctors he held as the only important one for medicine, not only as a matter of preference but in an absolute sense. The psychological discussions in his Textbook are brilliant in parts and he succeeded with them as it were unwittingly. He himself regards them as temporary stopgaps until experiment, microscope and test-tube permitted objective investigation.... [11] (pp. 853–855).

I think that the most important critique made by Jaspers of Kraepelin's nosology has to do with the use of the method of 'ideal types', which Jaspers borrowed from his mentor and friend, the sociologist Max Weber. The connection between Jaspers and Weber has also been insufficiently noted. In his discussion of the nature of meaning (subjective understanding, *Verstehen*, as opposed to *Erklären*, or the objective causal-empirical approach) in psychiatry, Jaspers writes in a footnote: 'The work of Max Weber was mostly responsible for my deliberate use of understanding as a method which would be in keeping with our great cultural traditions....This present book [*General Psychopathology*] (was) greeted as something radically new, although all I had done was to link psychiatric reality with the traditional humanities. Looking back now, it seems astonishing that that these had been so forgotten and had grown so alien to psychiatry' [11] (p 301–302).

Specific to nosology, Jaspers applies the ideal type method to the classification scheme of personality disorders, a scheme which he views as useful but not applying exactly to specific individuals in the real world. I would suggest that this analysis of ideal-types as the underlying method of psychiatric nosology might be extended to the entire current DSM system for all psychiatric disorders:

'If we think of a property as something lasting, understandable in its manifestations, in modes of reaction and expression and the general behavior of the individual, we are in process of developing a type. We form the property and all its consequences into a construct and viewing it as a whole recognize it as something obviously connected. If we then make one or several such properties the basis of a comprehensive totality and proceed to apply it to the person as a whole, noticing the meaningful connection between this and what the individual experiences and does, we are in the process of designing a personality-type. Such types remain ideal-types even if we have conceived them from our scrutiny of real people....They only occur in reality as approximate forms, as classical borderline cases....Thus the whole meaning of types makes it impossible for any one individual to be sufficiently characterized by any single type.' [11] (p. 434)

Jaspers also had other unique insights about Kraepelin's ideas and about diagnosis in general in his main chapter on nosology (chapter XII of Part IV of *General Psychopathology*, 'The synthesis of disease entities – Nosology', pp. 564–616). There Jaspers directly addresses the perspective of the empirical/biological school, led in his age by Kraepelin, and opposes the concept that psychiatric illnesses could be reduced to brain diseases, while recognizing the utility of the clinical empirical approach:

'There has been *no fulfillment *of the hope that clinical observation of psychic phenomena, of the life-history and of the outcome might yield *characteristic groupings *which would *subsequently be confirmed in the cerebral findings*, and thus pave the way for the brain-anatomists....The original question: are there only *stages and variants *of one unitary psychosis or is there *a series of disease-entities *which we can delineate, now finds its answer: *there are neither*. The latter view is right in so far that the idea of disease-entities has become a fruitful orientation for the investigations of special psychiatry. The former view is right in so far that no actual disease-entities exist in scientific psychiatry.' [11] (pp. 568–570, all italics in original text)

One might disagree in retrospect with that last statement, since at least some disease-entities, it can be argued, exist within the psychotic syndromes [12]. Earlier, in the Introduction to *General Psychopathology*, Jaspers also had commented:

'In the psychiatric assessment of a case...except in the case of well-known cerebral changes, diagnosis is the least relevant factor. If it is made the main issue, it will prejudge what ideally should emerge from the investigation. What matters is the process of analysis. The chaos of phenomena should not be blotted out with some diagnostic label but bring illumination through the way it is systematically ordered and related. Psychiatric diagnosis is too often a sterile running round in circles so that only a few phenomena are brought into the orbit of conscious knowledge.' [11] (p. 20)

But here in Part IV, Jaspers provides a classification scheme, heavily influenced by Kraepelin, remarkably similar to the nosology DSM-III and ICD-9:

'We have detailed knowledge of particular phenomena, of causal connections and meaningful connections, etc., but complex disease entities remain an endless, inextricable web. The individual configurations of disease are not like plants which we can classify in a herbarium. Rather it is just what is a 'plant' – an illness – that is most uncertain. *What *do we diagnose?...Diagnosis is expected to characterize in a comprehensive manner the whole morbid occurrence which has assailed the person and which stands as a well-defined entity among others....But however we devise [a diagnostic schema] we realize that it cannot work; that we can only make temporary and arbitrary classifications; that there are a number of different possibilities which account for the fact that different workers construct entirely different schemata; and that classification is always contradictory in theory and never quite squares with the facts. Why then do we keep on making this vain attempt? In the first place we want to see properly what this idea of disease-entity has *achieved *in respect to the *over-all picture *of existing psychic disorders, and particularly where we have failed because it is the basic and radical failure which makes us aware of the actual state of our knowledge. In the second place every *presentation of special psychiatry *requires some classification of psychosis at its base. Without some such schema it cannot order its material. In the third place we need a classification in order *to make statistical investigations *of a large case material.' [11] (p. 604, all italics in original text)

To translate: Jaspers is saying that psychiatric nosology is like botany; it is a clinical description of what we observe about persons with mental problems, much like botany is an observational description of the characteristics of plants. The larger question of "What is a mental illness?" is like asking the question "What is a plant?" It is much easier to describe specific plants than to fully explain the nature of being a plant (one might call it "planthood"). Jaspers is, perhaps, taking a stance here on the old philosophical debate about the particular versus the universal, and saying we should stick with the particular. He notes that nosology can never be definitive or absolutely valid (as is the case with everything in his philosophy), but he does *not *conclude, unlike many critics today, that psychiatric diagnosis is therefore useless. It has three utilities: 1. we need specific diagnoses by which to judge the larger question of overall mental illness; 2. we cannot engage in any specific aspect of psychiatry (I think this is what he means by "special psychiatry") without having some organizing schema for the whole (a general nosology; for Jaspers defining psychosis forms the basis for his nosology); 3. we need to make diagnoses reliably so we can engage in research (which is necessary for science, and Jaspers values science a great deal).

Jaspers goes on to provide the characteristics of an ideal nosology, features which DSM-III and IV would be seen as meeting rather poorly:

'An ideal schema would have to satisfy the following requirements: It must be such that any given case would have only one place within it and every case should have a place. The whole plan must have a compelling objectivity so that different observers can classify cases in the same way....We abandon the idea of disease-entity and once more have to bear in mind continually the various points of view (as to causes, psychological structure, anatomical findings, course of illness and outcome) and in face of the facts we have to draw the line where none exists. Such classification therefore has only a provisional value. It is a fiction which will discharge its function if it proves to be the most apt for the time. There is no 'natural' schema which would accommodate every case.' [11] (p. 605)

These are important caveats. Jaspers is in effect saying that diagnosis at its most supraordinate level (ideal types) has a basic and radical failure: it cannot be definitive or complete. Yet he goes on to acknowledge that psychiatry needs, both for practical and scientific purposes, to have a nosology. He then sketches a practical classification, largely derived from Kraepelin, as below, realizing, from the outset and quite explicitly that he is not carving nature at its joints, or identifying definitive disease-entities.

With those caveats, Jaspers proposes a nosology quite similar to DSM-III and ICD-9. He proposes dividing psychiatric conditions into three main groups [11]: Group I, 'Known somatic illnesses with psychic disturbances' (such as cerebral tumors, meningitis), approximates DSM's 'Axis III' which describes medical conditions that can influence psychiatric disorders. Group II, 'The three major psychoses' ('genuine epilepsy', schizophrenia, and manic-depressive illness), would correspond with the major mood and psychotic disorders on DSM's 'Axis I' of primary psychiatric conditions (with epilepsy now moved to Axis III since a cerebral basis has been established for it). Group III is the 'Personality disorders', which corresponds to DSM's 'Axis II', also defined as personality disorders. Heuristically, with the caveats given previously, Jaspers goes on to accept Kraepelin's definition of the distinction between schizophrenia and manic-depressive illness based on the outcome criterion as the main factor, i.e., invariably poor outcome with schizophrenia and frequent recovery with manic-depressive illness.

Jaspers' skepticism about nosology, however, does not end in nihilism. Because Kraepelin did not carve nature at its joints, Jaspers does not thereby reject Kraepelin's nosology. And Jaspers' acceptance of nosology is not merely practical; it is consistent with his entire philosophy that any human activity, including science, is never absolute in its knowledge; yet we can still have quite useful, even scientifically valid, knowledge nonetheless. Jaspers, in a word, was not a positivist, and his view of psychiatry in specific, and science in general, was more consistent with what philosophers of science today consider to be the nature of scientific work [13].

### Empathy and understandability

Readers familiar with Jaspers' work may have been most influenced by the section of *General Psychopathology *where Jaspers emphasizes empathy as a criterion for diagnosing delusions. Indeed, Jaspers' acceptance of the affective disorder/schizophrenia distinction in the nosology debate was in part based on the distinction between conditions with which one could empathize, those in whom meaningful connections could be made, and those which were not understandable. He felt that this distinction, based on the Erkalren/Verstehen distinction, would provide one of the few organizing principles for nosology:

'The most profound distinction in psychic life seems to be that between what is meaningful and allows empathy and what in its particular way is un-understandable, "mad" in the literal sense, schizophrenic psychic life (even though there may be no delusions). Pathological psychic life of the first kind we can comprehend vividly enough as an exaggeration or diminution of known phenomena and as an appearance of such phenomena without the usual causes or motives. Pathological psychic life of the second kind we cannot adequately comprehend in this way. Instead we find changes of the most general kind for which we have no empathy but which in some way we try to make comprehensible from an external point of view....The affective illnesses appear to us to be open to empathy and natural but the various types of "madness" do not seem open to empathy and appear unnatural.' [11] (pp. 577–578)

Perhaps it is this idea of Jaspers' that has attracted the most nosological attention, as it has become the source of the 'un-understandability' criterion for delusions. Numerous critiques of this view have been made, many of which I share. For the purpose of this paper I wish to put emphasis on Jaspers' underappreciated idea of ideal-types as being central to psychiatric nosology.

### Ideal types and psychiatric nosology

Much of Jaspers' nosology hinges on the concept of the ideal type, which is meant as a standard, or simplified, version of reality [14,15]. Let us take the example of historians, because this is where the concept was developed [16], yet realizing that everything that is said here can apply to the psychologist or the psychiatrist when faced with clinical aspects of treating patients. Historians observe certain aspects of a historical event. They then take those aspects that seem to be the most striking, those that are the most unique or interesting to them, and they abstract those aspects from the rest of the details of the event. Connected in the abstract, limited to the most salient aspects of the historical reality, historians thus create the ideal type for that event [14,15].

The point of the ideal type is not to directly correspond to reality, but to highlight certain aspects of reality that might otherwise get lost in the varying details of concrete reality. The ideal type is not seen as a scientific theory either, which changes as more and more information on the empirical details of concrete reality is gathered. The ideal type is itself the standard to which concrete reality is compared [15]. By using the word 'ideal', Weber did not mean that the ideal type is the best type, or better than concrete reality; he meant to emphasize the fact that it is an abstraction, a conceptualization made away from concrete reality [14].

A conceptual analogy to the ideal type is a ruler, by which objects are measured [15]. The ruler is not made based on empirical comparisons to reality. It simply is created by us, by humans, stipulated to be a certain length, and then used to measure real objects. Similarly, ideal types are concepts that are created by historians, and the facts of history are measured against them. The point of ideal types is to help us understand the meaning of those facts of history.

Ideal types might be seen as a hybrid between an essence (the necessary feature of something) and a tool (a measurement). One must acknowledge a large literature, especially in sociology, for and against the ideal type concept, so it is far from a settled simple notion. But it can at least be seen as one approach that, in the hands of Weber and Jaspers at least, seemed to bear some fruit.

Wiggins and Schwartz have suggested [17], and I wish to agree, that the DSM system of nosology in psychiatry can be seen as utilizing the same method. The diagnoses in DSM-IV are not 'real' entities; they are abstractions. (This is also relevant, probably, to internal medicine; I am not claiming that psychiatry is inherently different from the rest of medicine in this context). No single patient exactly meets the specific criteria of any diagnosis; every patient is uniquely different in some way. This reflects the concrete uniqueness of human existence, that aspect of human cultural reality which Weber and his predecessors so emphasized. Thus, the DSM diagnoses are not meant to directly correspond to clinical reality one hundred percent. Nor are they meant to represent explanatory theories of diagnoses, which are to be changed as more and more empirical evidence is gathered. In fact, this can happen, as noted previously, because the humanities can utilize the methods of the sciences to a certain degree. But, in principle, at root, the DSM criteria are stipulated by psychiatrists based on a consensus of their clinical experience, to best pick out the essence of various clinical situations, so as to best allow communication and research in a common language. This is an ideal type approach.

Thus, it is vain to criticize the DSM nosology for not corresponding to clinical reality. That is not its goal. The nosology is used to *categorize *clinical reality, not to copy it. Nor is it useful to criticize it for being abstract or for not being based completely on empirical evidence. Diagnoses cannot be established in psychiatry completely on the basis of empirical evidence, for the same reason that history cannot be comprehensively understood with facts and figures alone. Interpretations of meanings and motivations are unavoidable. Thus abstraction is necessary, unless one was to limit psychiatry to nothing but observation of the details of each clinical case, with no attempt at a more general level of understanding.

It is also no solution to criticize the DSM nosology based on the idea that clinical diagnostic categories (e.g., bipolar disorder) just have no correspondence to neurobiology and that we really need is to use psychopathological states (e.g., melancholia) or neuropsychological entities (e.g., working memory), and then correlate them with biological mechanisms [18]. This approach, so popular among the biologically minded, would be similar to saying, in 1900, that angina pectoris is a useless way to understand heart disease, and we need to instead start with radiating left arm pain. To reject syndromes of diagnosis for their composite symptoms is not a conceptual advance. One cannot prejudge the matter; it may well turn out that some of our clinical diagnoses, if honed better with more clinical research, well capture the biological mechanisms. But if we give up the clinical nosology research project, in favor of the biological *soup du jour*, we will never know.

Ultimately, the DSM nosology may lead to the recognition of some specific diseases, quantitatively defined in demonstrable abnormalities in brain structure or function or in genetic coding. At that point, the Verstehen-oriented ideal type approach will give way more completely to the Erklären-oriented empirical approach. However, given the uniqueness of human experience, it is likely that there will always be a role for some kind of ideal type approach in psychiatry.

### Where neo-Kraepelinian psychiatry has gone wrong

A critique of Kraepelin's nosology using the ideal type method derived from Jaspers (and Weber) becomes especially relevant when we examine the difference between Kraepelin himself and neo-Kraepelinianism, and the playing out of that difference in contemporary psychiatry.

As is well known, Kraepelin's nosology was focused on the major psychoses, with the main distinction between two primary illnesses, manic-depressive insanity and dementia praeocox. Kraepelin held that these two illnesses were disease-entities that they were due to biological abnormalities of the brain and body. Kraepelin was thus committed, as philosophers would put it, to a biological ontology for psychosis [1].

The neo-Kraepelinian school gave up this biological ontology, at least overtly [6], but extended Kraepelin's nosology beyond the major psychoses – to affective disorders (with non-psychotic unipolar major depressive disorder being very frequently diagnosed), anxiety disorders (especially generalized anxiety disorder, but also panic disorder, social anxiety disorder, and obsessive-compulsive disorder), personality disorders (with a score of different labels), and other conditions (e.g., attention deficit hyperactivity disorder – ADHD, post-traumatic stress disorder – PTSD). Some of these designations (like GAD) may have been mainly due to political compromises with psychoanalysts anxious to retain some categories that would capture their bread-and-butter label of 'neurotic depression' [19], but nonetheless the effect of the neo-Kraepelinian revisions of DSM-III and DSM-IV has been what van Praag has called 'nosologomania' – many disorders have been designated to 'exist' [20].

The problem in practice is that a DSM designation seems to translate, in the minds of many practitioners today, into the equivalent of a disease-entity. (I have discussed what characterizes a disease-entity as opposed to syndromes or symptoms elsewhere [14]; for current purposes, I mean by disease-entity an abnormality of the brain or body which might be caused in various ways but whose ultimate final pathway involves major biological abnormalities. For further discussion of this topic, see the above reference). In other words, where Kraepelin viewed his nosology as biologically committed to disease-entities, yet limited to severe psychotic conditions, contemporary neo-Kraepelinian American psychiatry denies a biological commitment in theory but practices as if there were biological commitments to over 300 DSM-defined entities [13], justifying pharmacological treatment [13]. Yet, in the tradition of scientific medicine dating from William Osler (as opposed to previous unscientific somatic approaches), physicians have been taught to use medications to treat diseases, not just symptoms [13,21]. (Again this is a key issue discussed in more detail in those texts; there I review the historical and conceptual background for supporting the view that scientific medicine is disease-oriented, not simply symptom-oriented). For many psychiatrists, DSM diagnoses are treated as proxies for diseases, and medications are duly prescribed, with not much in the way of intervening hesitation. As a result, symptoms hypertrophied into diagnoses appear to provide justification for medication prescription [21].

An example of this phenomenon may be the case of 'adult ADHD'. This condition was hardly described in the psychiatric literature until the 1990s. Even then most of the evidence suggested that it was extremely rare [22], with childhood ADHD either resolving in adulthood or changing its presentation into other adult psychiatric disorders. Yet, coincidental with the marketing of a drug in the United States (Strattera, atomoxitene) for presumed adult ADHD in 2002, a great increase of interest in the diagnosis of this condition has occurred [23], despite a relative paucity of evidence for the nosologic validity of this condition. For instance, proponents of the validity of adult ADHD often cite the National Comorbidity Survey analysis which found that 3% of the adult population met criteria for this condition, and that 36.3% of those individuals were retrospectively diagnosable with childhood ADHD [24]. However, 84.1% of those with presumed adult ADHD were also diagnosable with mood disorders (about equally bipolar disorder or unipolar depression) [24]. While this might be called comorbidity, it might also represent a lack of syndromal specificity, a major mark against diagnostic validity [25].

Concerns about nosologomania are especially relevant given some evidence of political and economic forces inside and outside of psychiatry that may be using our current neo-Kraepelinian framework for their own purposes. Critics have especially noted the influence of the pharmaceutical industry in 'selling sickness' or 'disease-mongering' [26], creating syndromes where none previously existed so as to create a marketplace for their drugs [19].

In my view, much of this diagnostic confusion stems from the neo-Kraepelinian attempt to stay 'neutral' ontologically. In practice, clinicians often make biological assumptions about treatment (e.g., using medications preferentially to psychosocial interventions) without scientific reasons to do so [13]. Instead, Jaspers' concept of ideal types, by reminding us of the abstract nature of our diagnoses and the fact that they do not *necessarily *correspond to natural disease-entities, may help contemporary psychiatry to better understand the conceptual nature of our diagnoses, getting a better handle on which ones likely are biologically-based disease-entities and which ones are not, and thereby making more appropriate decisions about which kinds of interventions are most appropriate. Further, this approach more honestly attends to our etiological ignorance (where present) about many psychiatric syndromes, and thus would lead to more cautious conclusions regarding pharmacological approaches to treatment. This perspective does not deny the utility of some pharmacological palliative treatment for symptoms, but it would give us conceptual clarity about what scenario holds (palliative symptom treatment or more definitive disease-oriented treatment), with practical consequences (less emphasis on medication treatment for superficial symptoms, and more for disease-oriented treatment).

## Conclusion

Emil Kraepelin's nosology has been reinvented, for better or worse. Today we need to better understand which diagnoses carry with them the implication of being biological disease-entities, thus requiring more emphasis on medication treatment, and which diagnoses are different (or at least about which we are relative ignorant regarding biologically-based etiology). The concept of ideal types, derived from Karl Jaspers, may help us reformulate our current neo-Kraepelinian nosology and thereby improve current psychiatric practice.

## Competing interests

In the past 12 months, Dr Ghaemi has received a research grant from Pfizer. Neither he nor his family hold equity positions in pharmaceutical corporations.
